# Horizontal Transmission of the Entomopathogen Fungus *Metarhizium anisopliae* in *Microcerotermes diversus* Groups

**DOI:** 10.3390/insects3030709

**Published:** 2012-08-08

**Authors:** Amir Cheraghi, Behzad Habibpour, Mohammad Saied Mossadegh, Mona Sharififard

**Affiliations:** 1 Department of Plant Protection, College of Agriculture, Shahid Chamran University of Ahvaz, Ahvaz 61357-831351, Iran; E-Mails: habibpour_b@scu.ac.ir (B.H.); ms.mossadegh@scu.ac.ir (M.S.M.); 2 Department of Medical Entomology, College of Health, Jundishapur University of Medical Sciences, Ahvaz, 61357-15751, Iran; E-Mail: sharififardm@yahoo.com

**Keywords:** termites, vector, recipient, biological control

## Abstract

An experiment was carried out in order to investigate fungal conidia transmission of *Metarhizium anisopliae* (Metschnikoff) Sorokin from vector (donor) to healthy *Microcerotermes diversus* Silvestri (Iso.: Termitidae) and determine the best donor/concentration ratio for transmission. After preliminary trials, concentrations of 3.1 × 10^4^, 3.9 × 10^5^, 3.2 × 10^6^ and 3.5 × 10^8 ^conidia mL^−1^ were selected for testing. The experiment was performed at three donor : Recipient ratios of 10, 30 and 50%. The highest mortality of recipient workers was observed after 14 days at the concentration of 3.5 × 10^8^ conidia mL^−1 ^and donor ratio of 50%. The mortality of recipient workers was less than 20% at all concentrations at a donor ratio of 10%. Our observations indicate social behavior of *M. diversus*, such as grooming, can be effective in promoting epizootic outbreaks in a colony. While the current results suggest good potential for efficacy, the use of *M. anisopliae* as a component of integrated pest management of *M. diversus* still needs to be proven under field conditions.

## 1. Introduction

Termites cause economic damage to timber, timber products, living plants and even man-made products such as foam insulation and plastics. Ecologically, termites are classified under three main groups: damp wood termites, dry wood termites and subterranean termites [1]. Subterranean termites form colonies that may contain thousands of individuals. Colonies of these termites are composed of workers, soldiers, nymphs and both primary and secondary reproductives. Workers are the most abundant caste, and they are responsible for many colony tasks, including foraging, construction and repairing the nest, feeding larva, soldiers, and other workers, and colony protection [2]. The nests of subterranean termites include simple galleries in wood or mounds which can be very complex and cryptic [3]. *Microcerotermes diversus* Silvestri (Iso.: Termitidae) is an extremely destructive structural wood pest and is considered to be the major species, in Iran, Iraq and Oman [4,5,6]. This species was identified as the major pest of date palms (*Phoenix dactylifera* L.) in Iran, Iraq and Saudi Arabia with a wide distribution [5,7]. Current management of subterranean termites in Iran mainly involves the application of a soil insecticide to reduce or isolate foraging populations [8]. Biological control is recognized as one of the alternatives to chemical pesticides [9]. Several international patents (or patent applications) involve the use of *Metarhizium anisopliae* (Metschnikoff)Sorokin in control of termites. Bio-Blast^TM^ Biological Termiticide, which is based on *M. anisopliae* isolate ESF-1 (EcoScience Corporation, NJ, USA), has been on sale in the United States following registration in 1994 [1]. There has been increasing interest in employing fungal pathogens to combat insect pests [10]. Entomopathogenic fungi use against termites can be beneficial because of its low environmental impact [9]. *M. anisopliae*, a hypocrealean Ascomycete, the causal agent of green muscardine disease of insects, is an important fungus in biological control of insect pests. The fungus is a facultative pathogen, which, as an entomopathogen, can infect insects [10]. Termites are considered to be good candidates for control with pathogens because they live in an environment conducive for the pathogen, which is a humid warm with minimal diurnal temperature fluctuations and crowded environment with considerable social interactions [1,11,12]. The use of entomopathogens to control termite colonies started >40 year ago when Lund (1966) patented fungal strains as biological control agents against subterranean termites [13,14]. Most of these studies focused on *Beauveria bassiana* (Balsamo) Vuillemin and *M. anisopliae* [15]. Both fungi are known to produce cuticle-degrading enzymes that facilitate percutaneous infection of the host insect without the need for oral consumption by the target organism [16]. Fungi exhibit qualities which can make them ideal for this infection strategy including a mild-acting nature, the ability to self-replicate and the ability of fungal conidia to be spread by termite social behavior [17]. Several studies examined fungal transmission in populations of termites [9,16,17,18,19,20]. There are no published reports on the transmission of *M. anisopliae* in populations of *M. diversus*. Our study was carried out to investigate fungal conidia transmission from vector to healthy termites in a colony and to determine the best vector/concentration ratio.

## 2. Experimental Section

### 2.1. Collection of Termites

Termites were collected by a method of planting blocks of beech (*Fagus orientalis* Lipsky) wood, 3 × 6 × 20 cm, in infested soil areas in Ahvaz (Khuzestan, Iran). In this manner worker termites were collected for this experiment.

### 2.2. Fungal Isolate

*M. anisopliae* strain DEMI 001 (Iranian Research Institute of Plant Protection collection), isolated from *Rhynchophorus ferrugineus* Olivier (Coleoptera: Curculionidae) in Saravan (Iran), was used. The fungus was cultured on Sabouraud Dextrose Agar with 1% yeast extract. Petri dish cultures were incubated at 28 ± 1 °C and 85 ± 5% relative humidity. Two-week-old, sporulated cultures were used for testing.

### 2.3. Preparation of Fungal Suspension

Two-week-old conidial suspensions were prepared by lightly scraping the surface of fungal cultures with a sterile scalpel and suspending the conidia in 100 mL distilled sterile Tween 80^®^ 1% solution. The conidial concentration of the suspensions was determined with a haemocytometer.

### 2.4. Experiment

**Figure 1:**
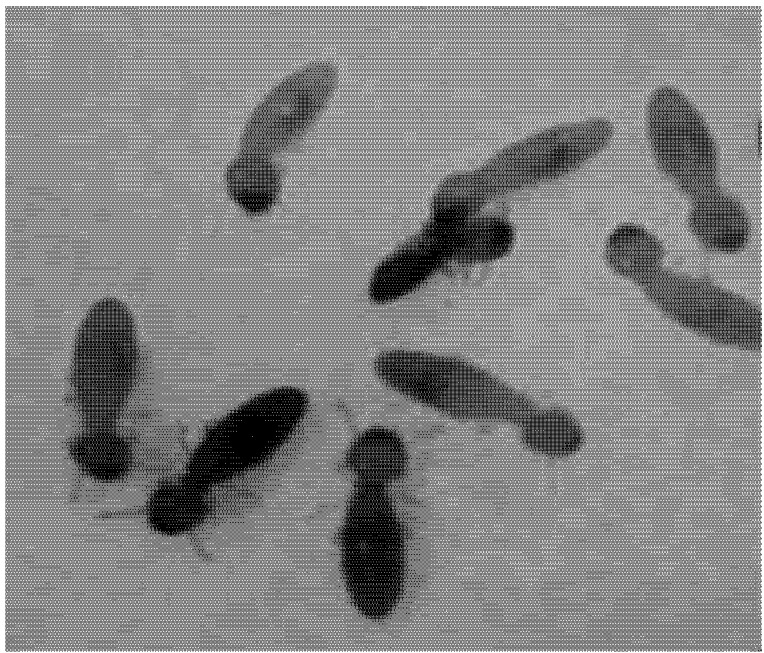
Placing blue vector termites (dark termites) among healthy termites (white termites).

The pathogenicity and viability of *M. anisopliae* was demonstrated on *M. diversus* through the Koch test. According to the preliminary trials ‘determination of concentration test’, concentrations of 3.1 × 10^4^, 3.9 × 10^5^, 3.2 × 10^6^ and 3.5 × 10^8 ^conidia mL^−1 ^were selected for testing. The experiment was performed in three vector (donor) : Recipient (target) ratios of 10, 30 and 50%. Each treatment included four replications of 50 workers each. Termites that had been designated as vectors were dyed blue by being allowed to feed on filter paper treated with 1 mL of 0.25% Nile Blue solution. Next, the termites were immersed in each concentration suspension. They were then placed into the population as vectors (Figure 1). Control vector termites were immersed in distilled water containing Tween 80. Each test unit consisted of a plastic Petri dish (9 cm diameter) containing No. 1 grade Whatman filter paper that was sprayed with 1 mL distilled water. Mortality was recorded for 14 days after introduction of the vector termites. Termite cadavers that were blue were not removed from Petri dishes to allow the fungus to spread in the group.

### 2.5. Statistical Analysis

Mortality data were subjected to angular transformation and analyzed using analysis of variance (ANOVA). PROC MIXED was used in the SAS software (SAS Institute, 2000). Means were compared by using the least signiﬁcant difference (LSD) at α = 0.05 after ANOVA (SAS Institute, 2000). Corrected mortality from fungal treatments was calculated using Abbott’s formula (Abbott, 1925).

## 3. Results and Discussion

**Figure 2:**
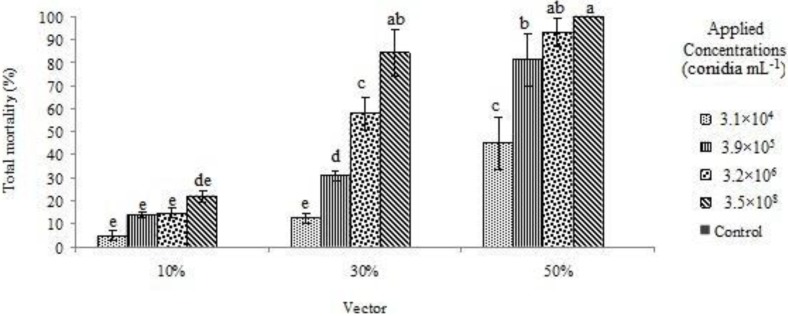
The level of total mortality for different treatment combinations after 14 days.

Figure 2 shows total mortality (vector and recipient) for the different conidial concentrations and proportions of vectors to recipients. The highest mortality was observed after 14 days at a concentration of 3.5 × 10^8^ conidia mL^−1^, with a 50% vector ratio. Total mortality was less than 30% at all concentrations, when the percentage of vectors was 10%. When the conidial concentrations were 3.5 × 10^8^, 3.2 × 10^6^ and 3.9 × 10^5^ conidia mL^−1^ with a vector ratio of 50% or when the concentration was 3.5 × 10^8^ conidia mL^−1^ with a 30% vector ratio, the total mortality was larger than 80%. There was no significant difference between a concentration of 3.5 × 10^8^ conidia mL^−1^ with 50% and 30% vector ratios, and a concentration of 3.2 × 10^6^ conidia mL^−1^with a 50% vector ratio. Total mortality indicated no significant difference between the concentration of 3.9 × 10^5^ conidia mL^−1^ at 50% vector ratio and the 3.5 × 10^8^ conidia mL^−1^ concentration at 30% vector ratio and the 3.2 × 10^6^ conidia mL^−1^ concentration with the 50% vector ratio. In general, total mortality was significantly different at all concentration levels between the 50, 10 and 30% vector ratios at each conidial concentration. Total mortality data also indicated significant differences at all levels between the 30 and 10% vector ratios, with mortalities at the 30% vector ratio being consistently higher than at the 10% vector ratio.

**Figure 3:**
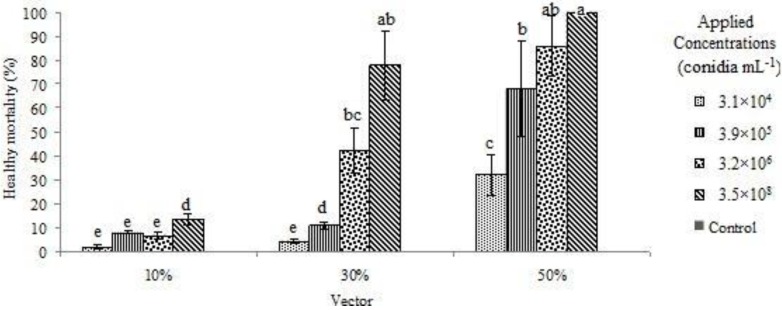
The level of healthy (recipient) workers mortality for different treatment combinations.

Figure 3 shows the mortality of the recipient termites only at the different concentrations and proportions of vectors to recipients. The highest mortality level was observed at 14 days post-treatment at a concentration of 3.5 × 10^8^ conidia mL^−1^ and with a 50% vector ratio. At all concentrations, the mortality of recipient workers was less than 20% when the vector ratio was 10%. When the conidia concentrations were 3.5 × 10^8^ and 3.2 × 10^6^ conidia mL^−1^ with a 50% vector : Recipient ratio, mortality of the target termites was more than 80%. When the concentration was 3.9 × 10^5^ conidia mL^−1^ with a 50% vector: Recipient ratio, or when the concentration was 3.5 × 10^8 ^conidia mL^−1^ with a 30% vector ratio, mortality of workers was more than 65%. There were no significant differences among mortalities when the concentration was 3.5 × 10^8^ conidia mL^−1^ with 50 and 30% vector ratios and the concentration of 3.2×10^6^ conidia mL^−1^ with 50% vector ratio. The recipient mortality showed no significant differences among the treatments of 3.9 × 10^5^ conidia mL^−1^ with 50% vector ratio, 3.5 × 10^8^ conidia mL^−1^ with 30% vector ratio and 3.2 × 10^6^ conidia mL^−1^ with a 50% vector ratio. In general, the mortality of recipient termites showed significant difference between the 50% vector ratio and the 10 and 30% vector ratios at the same conidia concentrations. In addition, the mortality of recipient termites was significantly larger at all concentrations with the 30% vector ratio than with the 10% vector ratio.

The time to 50 and 90% mortality (LT_50_ and LT_90_, respectively) reflected the overall mortality data. Table 1 shows LT_50_ and LT_90_ values for the different vector ratios at different conidia concentrations. The lowest LT_50_ value was 2.33 days and the lowest LT_90_ value was 12.86 days; both at a concentration of 3.5 × 10^8^ conidia mL^−1^ and 50% vector ratio (df = 12, F = 28.69, *p* < 0.0001). The largest LT_50_ and LT_90_ values were obtained with a 10% vector ratio. Our results show that the LT_50_ and LT_90 _were reduced as the vector ratios increased. They were also reduced by increasing the number of vectors at each concentration.

**Table 1:** The level of LT_50_ and LT_90_ for different treatment combinations.

Concentration	Vector (%)	LT_50_(day)	LT_90_(day)
(Conidia mL^−1^)		(95% Fiducial limits)	(95% Fiducial limits)
3.1 × 10^4^	10	-*	-
	30	-	-
	50	19.5 (17–23)	-
3.9 × 10^5^	10	-	-
	30	30 (21–55)	-
	50	7.27 (6.4–8.29)	27.81(21.22–41.63)
3.2 × 10^6^	10	-	-
	30	12.12 (10.32–15.04)	-
	50	4.68 (3.51–5.85)	23.49(15.96–48.05)
3.5 × 10^8^	10	-	-
	30	6.23 (4.51–8.58)	47.32(24.77–215.03)
	50	2.33 (1.20–3.32)	12.86(8.82–26.99)

* The high values of LT_50 _and LT_90 _are not reported.

Mechanical transmission of the infectious conidia by workers exposed to the fungi outside of the nest is very important for these fungi to be effective against termites. Our research offers a better understanding of the contamination process and the conidia transfer between termites.

Our study showed that vectors are able to transfer fungi successfully to recipient workers at high concentrations of conidia suspensions and a high ratio of vectors in *M. diversus*. Vector : Recipient ratios of 30 and 50% were more effective than the 10% vector ratio, especially with high concentrations of conidia. Due to lower and slower mortality_, _treatment of vector workers with low concentrations of conidia suspension and a vector : Recipient ratio of 10% were not promising. Results from our present research confirmed results reported by Grace and Zoberi (1992), Bayon *et al.* (2000), Myles (2002) and Wright *et al*. (2002) with other termite species [9,16,17,21].

Desyanti (2010) applied the entomopathogenic fungus *Myrothecium roridum* Tode ex Steudel and *Metarhizium* sp. with vector ratios of 5, 10 and 15% using *Coptotermes gestroi* Wasman (Blattodea: Rhinotermitidae) [20]. Desyanti *et al*. (2009) also reported on the transmission of entomopathogenic fungi *Metarhizium brunneum* Petch and *M. roridum* in *Cryptotermes* sp. (Blattodea: Kalotermitidae) [19]. They used vector ratios of 10, 20, 30, 40 and 50% and an inoculum concentration of 10^7^ conidia mL^−1^. They showed that there was a correlation between the proportion of vector to recipient termites and application period with mortality. Our findings are consistent with those results. Overall, concentrations should be used that cause higher mortality in a shorter timeframe. These results may be beneficial in field applications because social behaviors of termites, such as grooming and trophallaxis, may be effective in creating epizootic conditions. At the same time, the conidia transmission may also reduced by the cleaning behavior of nestmates in large colonies under field conditions.

Because termites live at high densities, horizontal transmission is extremely important for the fungal application in the field. Horizontal transmission between individuals of the same species (autodissemination) can occur through direct contact between contaminated and uncontaminated individuals or indirectly via conidia that have been deposited on the substrate [22,23,24]. In addition, a positive relationship between insect movement and the transmission of entomopathogenic fungi has been observed in a number of systems [25]. Cory and Hoover (2006) stated that insects and entomopathogenic fungi were under opposing selection pressures. Insects gain a selective advantage from detecting and avoiding fungal pathogens, while the successful infection to an insect by an entomopathogen requires contact between the host and the pathogen. The behavior of insects can inﬂuence whether contact is made, with change in activity increasing or decreasing the likelihood of infection [26].

On the other hand Chouvenc *et al.* (2008) stated that termites might gain a selective advantage in detecting the risk of attack from entomopathogenic fungi and by responding via behavioral avoidance or through post-contact responses such as grooming. These responses may reduce the efficiency of the fungal infection in the field. In contrast, fungal pathogens could gain an advantage by attracting or remaining invisible to host insects [27]. The ability of insects to detect and respond to entomopathogenic fungi within the order Hypocreales has been widely assessed, with reports of avoidance of fungi by species within the Coleoptera, Isoptera, Hemiptera and Orthoptera [1,18,27,28,29,30,31]. Yanagawa *etal*. (2010) noted that *C. formosanus* protects itself from entomopathogenic fungus by mutual grooming behavior. These termites remove foreign organisms, such as fungal conidia, from the body surfaces of their nestmates. In addition, they found that *C. formosanus* could detect fungus species via odor [32].

Generally, social interactions such as allogrooming, proctodeal and stomodeal trophallaxis, coprophagy and cannibalism of diseased or injured nestmates increase the dissemination of pathogens. These behaviors may actually also decrease infection risk. Termites avoid contaminated areas or infected nestmates, and may move to a new nest after an encounter with pathogenic or parasitic microbes.

The effect of the hygiene behaviors of termites towards entomopathogenic fungi should be considered in future research. It is important to find a way of weakening such defensive mechanisms. If these protective mechanisms were made less effective, occurrence of epizootics should increase. The use of insecticides, such as imidacloprid, in combination with an entomopathogen can be an effective strategy. These insecticides should be compatible with disease agents and applied in the form of sublethal concentrations to weaken behavioral responses of termites and thus increase the possibility of disease transmission [33]. The use of sub-lethal concentrations of imidacloprid in *R. ﬂavipes*, affected hygiene function (e.g., grooming), resulting in increased infection with *B. bassiana* [34].

## 4. Conclusion

Direct inoculation of fungi onto the body surface of vector termites was used in this study. Further work is recommended to examine the effect of fungus transmission from mycotic termite cadavers within the rest of the colony. Because termite contact with pathogens is important for creating a fungal epizootic among the population, processes associated with this phenomenon should be examined closely. It seems that the successful microbial control of termites requires consideration of not only the infectivity and pathogenicity of the agent, but also the termite protective behaviors. If solutions can be found to overcome the termite activities which reduce the fungal epizootics in a colony, the probability of successful biological control will be greater. In subsequent research, virulence of this pathogen can be compared with other isolates. In addition, the optimum condition for occurrence of maximum pathogenicity of entomopathogenic fungus can be investigated. Using *M. anisopliae* as a component of integrated pest management of *M. diversus* still needs to be proven in field application.
